# Study on the Regeneration-Cycle Mechanism of Cu-BTC@MWS Composites Following Mercury Adsorption

**DOI:** 10.3390/polym17182474

**Published:** 2025-09-12

**Authors:** Feng Wang, Yue Yu

**Affiliations:** 1 Ningxia Energy Aluminium Linhe Power Branch, State Power Investment Corporation Limited, Yinchuan 750411, China; 18995390198@189.cn; 2 College of Economics and Management, Taiyuan University of Technology, Taiyuan 030024, China

**Keywords:** Cu-BTC, mercury removal, density functional theory, molecular structure, regeneration mechanism

## Abstract

Coal-fired power plants, as the largest source of human-made mercury emissions, often lack specialized mercury emission control devices. Therefore, developing cost-effective adsorbents and studying their regeneration properties are highly important for mercury removal from flue gas. In this study, the regeneration efficiency and stability of a composite material made from polymetallic Fe/Cu-doped modified biochar combined with the MOF material Cu-BTC were investigated. Based on the analysis of microscopic characteristics, the molecular structure of the regenerated composites was modeled, and the adsorption and regeneration process of Hg^0^ on their surface was simulated using density functional theory. This helped uncover the underlying mechanisms of mercury removal and regeneration. The results indicate that the optimal regeneration temperature and atmosphere were 350 °C and 5% O_2_, resulting in the formation of a derived carbon material. The regeneration efficiency reached 92% of that of the original mercury adsorption capacity, and over 80% efficiency was maintained after 10 regeneration cycles. The regenerated samples adsorbed Hg^0^ through the combined action of surface metal oxides, the metal element Cu, and oxygen-containing functional groups.

## 1. Introduction

Mercury (Hg), entering the environment due to natural and anthropogenic reasons, is carcinogenic, teratogenic, mutagenic, has other toxic effects, is extremely hazardous to human health [1,2], and has gradually come to the public’s attention. Mercury released during the flue gas emission process of coal-fired power plants in China is the main source of atmospheric mercury, which mainly exists in the form of oxidized mercury (Hg^2+^), particulate mercury (Hg^p^), and singlet mercury (Hg^0^). Among them, Hg^2+^ and Hg^p^ can be removed by precipitators and wet flue gas desulfurization (WFGD) devices, whereas Hg^0^ is challenging to remove using the existing flue gas purification devices because of its insolubility in water and volatility [3,4,5]. With increasingly stringent environmental emission standards, mercury pollution prevention and control have been incorporated into the long-term planning of power companies. The coal-fired units at the Linhe Power Generation Branch of China Power Investment Corporation’s Ningxia Energy Aluminium Industry have relatively high mercury and mercury compound emission concentrations, approaching 0.03 mg/m^3^. According to relevant technical guidelines issued by the Ministry of Environmental Protection, coal-fired power plants are encouraged to adopt technologies such as adsorbent injection to control mercury emissions. At present, the adsorbent injection method, capable of being used in conjunction with electrostatic precipitators and baghouses, is a promising technology for mercury emission control, so the development of cost-effective adsorbents has become an urgent problem.

The Cu-BTC materials studied at this stage are widely used in adsorption, separation, and photocatalysis because of their good stability, large specific surface area, and exposed adsorption sites created by unsaturated metal-centered copper dimers [6,7]. Developing cost-effective mercury adsorbents from biomass as raw material is an important technical approach for converting waste into less toxic forms. Using biomass to remove combustion pollutants can address the limitations of biomass’s calorific value and the lower efficiency of individual use, making it a key strategy for the effective utilization of biomass. This area of research has seen wide activity internationally [8,9,10]. The modified biochar prepared from biomass is rich in oxygen-containing functional groups and metal sites. After pretreatment, it can generate a sufficient number of active sites and functional groups for coordinating with MOFs, making it possible to combine Cu-BTC with modified biochar to create a Cu-BTC-based composite adsorbent for removing mercury from flue gas pollutants.

The regeneration and reuse of adsorbents are very important for conserving resources, lowering the cost of mercury removal, and enhancing the economic efficiency of adsorbents. The main methods of regenerating mercury adsorbents include low-temperature plasma regeneration [11], wet elution regeneration [12], and thermal desorption regeneration [13]. Thermal desorption regeneration is suitable for all types of adsorbents and is relatively easy to operate. Currently, the regeneration process for mercury adsorbents mainly involves heating the deactivated adsorbent to high temperatures to decompose and release the mercury compounds deposited on the surface, thereby exposing the active sites that are covered or inactivated.

San Miguel et al. [14] studied the regeneration process of waste activated carbon used for water treatment and found that the specific surface area of activated carbon recovered 64% to 93%, while the pore volume of micropores recovered 77% to 97% after the adsorbent was treated at 800 °C in an N_2_ atmosphere for 60 min. The regeneration efficiency could be further improved by activating the carbon with water vapor during the process. However, after regeneration, the porosity and BET surface area of the activated carbon decreased significantly, leading to a notable reduction in its capacity to adsorb small organic molecules. Zhou et al. [15] investigated the mercury removal characteristics of Ce-modified Fe–Mn magnetic adsorbent through thermal desorption regeneration and observed that the mercury removal efficiency of the regenerated adsorbent increased and then declined with rising regeneration temperature. The Fe_6_Mn_0.8_Ce_0.2_O_y_-R adsorbent, after undergoing thermal regeneration at 500 °C, maintained a 95.75% mercury removal rate after 150 min of adsorption. Additionally, the presence of O_2_ replenished the lattice oxygen consumed during mercury removal, aiding in the regeneration of the adsorbent. The Fe_6_Mn_0.8_Ce_0.2_O_y_ adsorbent demonstrated excellent mercury removal performance after six cycles of mercury adsorption at 500 °C under an air atmosphere. In recent years, the use of agricultural waste to remove flue gas pollutants has gained widespread attention and application. It was found that walnut shells were more effective in removing mercury compared to corn husks, coconut shells, and cotton stalks (Figure S1). The output of walnut shells in Shanxi ranked second in China, so walnut shells were used as the raw material [16].

Currently, research on the regeneration performance of the composite adsorbent and its regeneration mechanism remains at the macroscopic level. There are fewer comprehensive studies on microscopic characteristics, and the related mechanisms are not fully explained. Therefore, this study examined how the coupling between O_2_ concentration and temperature affects regeneration performance and identified the optimal regeneration conditions using experimental methods combined with various characterization techniques. Additionally, based on density functional theory (DFT), theoretical calculations were performed on the adsorption process of Hg^0^ on the surface of regenerated samples to investigate the regeneration mechanism and key action mechanisms in more detail.

## 2. Materials and Methods

### 2.1. Sample Preparation

In this paper, Fe/Cu modified biochar samples (MWS) were prepared using the sol–gel method and co-precipitation method, based on the process described by Wang et al. [17]. Cu-BTC was prepared through the solvothermal method [18], and Cu-BTC@MWS samples were synthesized via in situ growth. Refer to Supplementary Materials Part S2 for detailed information on the preparation process.

### 2.2. Characterization

An ultimate analysis was conducted to ascertain the elemental composition of the samples using an organic elemental analyzer (Elementar, Frankfurt Meck, Darmstadt, Germany) and an ICP analyzer (Thermo Fisher, Waltham, MA, USA). The crystalline phase structure of the samples was characterized using a D8 ADVANCE series X-ray diffractometer (Bruker, Berlin, Germany) at a scanning rate of 10°/min. The chemical characterization of the surfaces was performed using a Nicolet iS20 Fourier Transform Infrared Spectrometer (Thermo Fisher, Waltham, MA, USA), operating within a scanning range of 400–4000 cm^−1^, with a resolution of 4 cm^−1^, and 16 scans. The microscopic morphology was examined utilizing an electron microscope (Hitachi, Tokyo, Japan), and the pore structure was analyzed via a Specific Surface and Porosity Analyzer (Micromeritics, Norcross, GA, USA). Nuclear Magnetic Resonance (NMR) experiments were conducted on a 600 MHz superconducting NMR instrument (Bruker, Berlin, Germany) equipped with a 3.2 mm dual resonance MAS probe, using a pulse width of 4 μs and a pulse delay of 1 s. The thermal stability of the compound adsorbent was assessed using a comprehensive thermogravimetric analyzer (HCT-1, Beijing, China).

### 2.3. Hg^0^ Fixed-Bed Adsorption/Regeneration Experiment System

**Figure 1:**
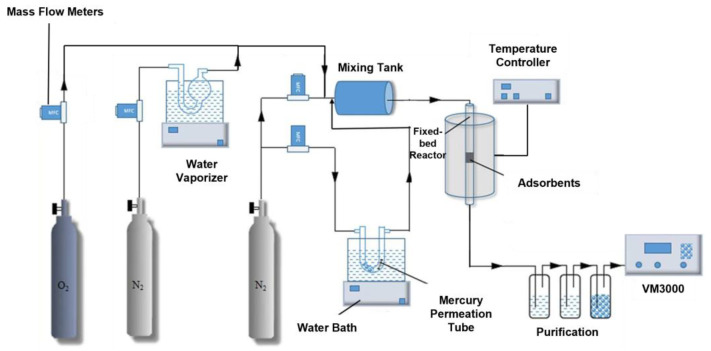
Fixed-bed mercury adsorption/regeneration experimental system.

In this experiment, a fixedbed mercury adsorption/regeneration test system (Figure 1) was used to perform the mercury adsorption of the adsorbent, consisting of a gas distribution system, a mercury vapor generator, a fixed-bed adsorption device, a VM3000 mercury continuous on-line monitor (made by MI Company, Karlsfeld, Germany), and a tail gas treatment device. The specific adsorption experimental procedure is described in Part S3 of the Supplementary Materials.

During the optimization of regeneration conditions, first, 0.1 g of deactivated adsorbent sample was weighed and placed in the quartz glass tube of the tube furnace, with N_2_ (800 mL/min) introduced as a protective gas. The tube furnace was heated at a rate of 15 °C/min and maintained at a constant temperature for 1 h. A total of 5% O_2_ gas by volume was introduced, and the N_2_ flow was adjusted to keep the total gas flow constant. The total flow was regulated by adjusting the N_2_ flow, and the temperature was increased to 300 °C, 350 °C, and 400 °C, with each held at a constant temperature for 2 h before cooling to room temperature to obtain the activated samples. Similarly, 350 °C was chosen as the regeneration temperature, and the process was repeated with varying O_2_ volume concentrations of 3%, 5%, and 7%. The molecular structure of the composite adsorbent collapsed when heated to 350 °C in 5% O_2_, forming the Cu-BTC-based modified biochar composite adsorbent-derived carbon material, labeled as R-Cu-BTC@MWS.

### 2.4. Model Construction and Simulation Calculation Method

In this study, based on the mercury-adsorption cycle-regeneration experiment, the R-Cu-BTC@MWS sample was selected as the research focus to investigate the molecular structure formation and the mechanism of cyclic regeneration during Hg^0^ adsorption. The molecular microstructural features of the regenerated samples were primarily obtained through the results of ultimate analysis, FTIR, and NMR spectroscopy. A monomer model of the molecular structure was built, and the NMR spectra of the constructed 2D molecular configurations were fitted using ChemBioOffice Ultra 14 software. The model was validated by comparing it with the measured experimental values, calculated based on the integral area of different carbon groups [19]. Using the 2D molecular configuration, Gaussian 16 software was employed to construct a monomer model, perform structure optimization (using a PBE1PBE generalized-gradient functional), with the 6-311G+(d, p) basis set for nitrogen and hydrogen, def2svp basis set for carbon and oxygen, and SDD pseudo-potential for iron and copper [20]. The simulated molecular structure was used to analyze the regeneration mechanism of Hg^0^ adsorption on the sample surface.

## 3. Results

### 3.1. Adsorption and Regeneration Cycle Characteristics

**Figure 2:**
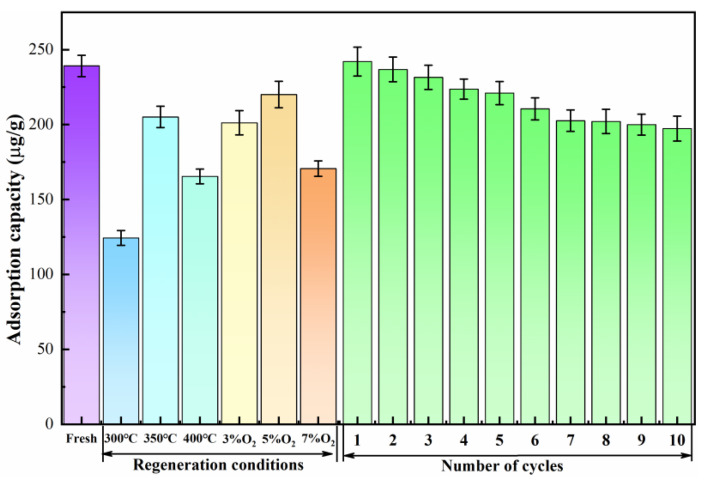
The effect of regeneration temperature and O_2_ volume on regeneration characteristics.

The mercury compounds existing on the surface of the adsorbent are mainly released by the high temperature conditions during the regeneration process, facilitating the re-exposure of the occupied functional groups and adsorption sites and repairing the inactivated adsorption sites by using O_2_. Therefore, the regeneration temperature and O_2_ concentration are important influencing factors in the regeneration process [21]. To obtain the corresponding influence mechanism, the regeneration and mercury removal characteristics were investigated for the Cu-BTC@MWS samples obtained in the previous section (Figure 2).

When the O_2_ concentration was 5%, the Hg^0^ adsorption capacity of the regenerated samples showed a tendency to increase and then decrease as the regeneration temperature rose. The highest Hg^0^ adsorption capacity was achieved at 350 °C. This is because the regeneration temperature not only affects the decomposition and release of mercury on the surface of the deactivated adsorbent but also directly influences the stability of the active components [22], making temperature a key factor in the mercury removal performance of deactivated and regenerated samples. At 350 °C, the entire framework of the deactivated Cu-BTC@MWS sample collapsed, forming the derived carbon material R-Cu-BTC@MWS. This process facilitated the release of surface-bound mercury compounds and exposed active sites such as metal centers and functional groups. Meanwhile, in an appropriately balanced O_2_ atmosphere, O_2_ reacted with the carbon to create new oxygen-containing functional groups, thereby effectively enhancing the mercury removal efficiency of the regenerated samples [23]. However, at 300 °C, the mercury removal efficiency of the regenerated samples was only 52% of that of the fresh samples. This was because the organic ligands began to decompose at this temperature, and the diffused O_2_ could not promote pore development or the replenishment of oxygen-containing functional groups. Additionally, under the presence of O_2_, the mercury compounds originally deposited in the inactivated samples were not fully decomposed and released, leading to a lower mercury adsorption capacity in the regenerated samples. When the regeneration temperature reached 400 °C, the adsorption efficiency dropped by 19.7% compared with that at 350 °C. This decline was due to the intense oxidation and even combustion reactions caused by the O_2_, as well as the collapse and accumulation of the internal structure at this temperature. The pore richness decreased dramatically, ultimately impairing the diffusion of Hg^0^ into the regenerated samples.

Based on the optimal regeneration temperature of 350 °C, the effect of O_2_ volume on the adsorption performance of the regenerated samples was further investigated. It was found that the influence of O_2_ volume on regeneration performance resembled that of regeneration temperature, with the Hg^0^ adsorption capacity of the samples increasing and then decreasing as O_2_ volume in the regeneration atmosphere was raised. When O_2_ volume was at 3% and 5%, the mercury removal performance improved, with the maximum mercury adsorption capacity reaching 219.97 μg/g at 5% O_2_, which is up to 92% of that of fresh samples. This indicates that introducing O_2_ during regeneration promotes some activation reactions in the sample. When O_2_ levels are low, O_2_ significantly affects the development of pore structure and surface chemical properties. As O_2_ diffuses into internal pores, it facilitates the precipitation of uncracked volatile components and tars, enhancing pore development [24]. Additionally, O_2_ replenishes oxygen atoms in functional groups degraded during Hg^0^ adsorption or reacts with unsaturated carbon atoms to form new carboxyl, carbonyl, or carbon–oxygen complexes, leading to activation and creating reactive sites for adsorption. The O_2_ can also replenish lattice oxygen used during Hg^0^ oxidation, repairing oxidized sites. However, when the O_2_ concentration reaches 7%, the process shifts into a vigorous oxidative combustion, depleting metal active sites and causing inactivation, which significantly diminishes the mercury removal performance.

The regeneration stability of the R-Cu-BTC@MWS samples was investigated based on the optimal regeneration conditions. It was observed that after one regeneration cycle, the efficiency of R-Cu-BTC@MWS reached 92% of that of new samples. However, the overall regeneration efficiency gradually declined with more cycles. This occurred because the sample was repeatedly heated to 350 °C in an oxygen-containing atmosphere, causing the oxygen-containing functional groups present on its surface to undergo cleavage or desorption. Additionally, after multiple regenerations, some adsorbents become difficult to completely remove due to reoxidation, causing residual adsorbents to accumulate and eventually impair the mercury removal performance of the samples.

### 3.2. Microscopic Characterization

#### 3.2.1. Weight Loss Characteristics

**Figure 3:**
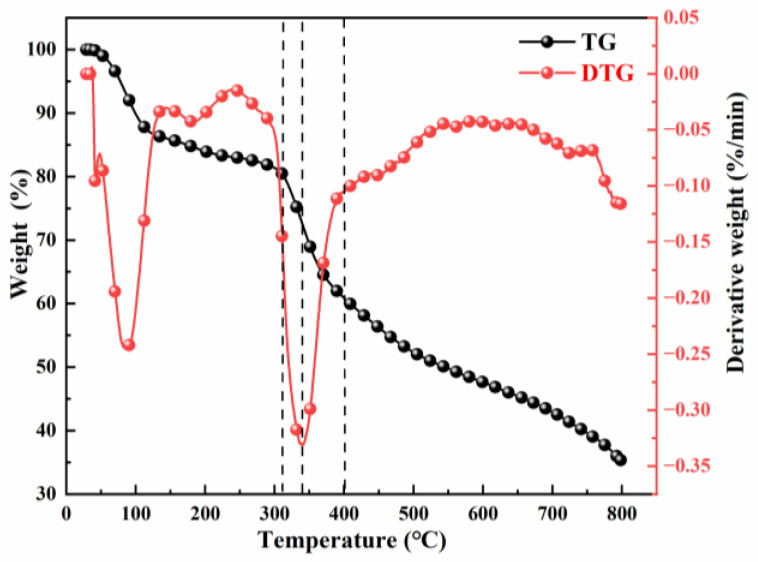
Weight loss characteristics.

To examine how the regeneration process affects the microscopic features of the samples, the thermal weight loss of the composite adsorbent Cu-BTC@MWS was analyzed, and the results are shown in Figure 3. It was found that when heated from 50 °C to 100 °C, the sample lost about 10% of its mass. The first weight loss peak appeared at around 80 °C, mainly due to the evaporation of free water in the pores. When the temperature increased from 100 °C to 300 °C, the mass loss reached about 19%, caused by the oxidation of the composite’s carbon component through doping of the modified biochar [25]. As the temperature rose from 300 °C to 350 °C, the mass loss climbed to approximately 33%. The peak of maximum weight loss rate appeared around 350 °C. At this point, the coupling bond between Cu-BTC and the modified biochar was broken, decomposition of the organic ligands in Cu-BTC occurred, and the main carbon framework of the modified biochar, along with surface functional groups, decomposed. Metal oxides also decomposed, leading to the breakdown of the composite’s overall structure. Consequently, the composite collapsed structurally, producing derived carbon materials. When the temperature was increased further to 400 °C, the mass loss reached about 42%, and the sample’s structure was essentially destroyed, with structural stacking effects observed.

#### 3.2.2. Ultimate Analysis

The samples were analyzed by ultimate analysis, and the resulting chemical components and their contents are shown in Table 1. From the previous section, since the R-Cu-BTC@MWS sample is a highly heterogeneous mixture formed by high-temperature calcination of a Cu-BTC-based modified biochar composite adsorbent, the elemental compositions of the two samples were similar. Additionally, compared to the composite material, the carbon content in the R-Cu-BTC@MWS sample increased, while the oxygen content decreased, leading to a lower O/C ratio. This change is attributed to the collapse of the original framework structure of the Cu-BTC material and the decomposition and shedding of organic ligands during regeneration and heating. The increase in the H/C ratio is due to the overall molecular weight of R-Cu-BTC@MWS being much smaller than that of the composite adsorbent. To ensure the diversity and scientific accuracy of the molecular structural units in the R-Cu-BTC@MWS mixture, its molecular weight was estimated to be around 1000 [26]. The initial molecular formula was proposed as C_46_H_26_O_10_N_2_Fe_2_Cu, with *M*_r_ = 942 by elemental ratio calculations. Both the structural integrity of the regenerated composite adsorbent samples and the requirements of the simulation process were satisfied.

#### 3.2.3. Crystal Phase Structure and Surface Chemical Characteristics

**Figure 4:**
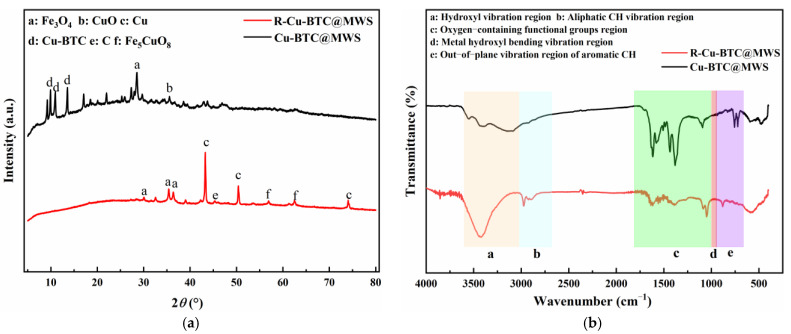
XRD and FTIR results. (**a**) Crystalline structure; (**b**) Surface chemical characteristics.

The crystal phase structures of Cu-BTC@MWS and R-Cu-BTC@MWS are shown in Figure 4a. Among them, the diffraction peaks attributed to Fe_3_O_4_ appeared at 30.04°, 35.38°, and 36.44° as observed from the R-Cu-BTC@MWS pattern. Compared to the Cu-BTC@MWS samples, the diffraction peaks corresponding to Fe_3_O_4_ were significantly increased, indicating that oxygen oxidation converted some Fe^2+^ to Fe^3+^ and that metal oxides formed during the creation of the derived carbon materials. Diffraction peaks attributed to graphitic carbon appeared at 45.46°, with a notable increase in the degree of graphitization compared to fresh composite samples. Peaks corresponding to Cu^0^ appeared at 43.26°, 50.34°, and 74.1°, showing that Cu^2+^ in the composite was reduced to Cu^0^ during high-temperature regeneration. Peaks related to Fe_5_CuO_8_ material appeared at 56.96° and 62.44°, which was a type of composite oxide with dynamic oxygen storage and release abilities, indicating that the regeneration process promotes the formation of oxygen-storing solid solutions in the samples. This process supplies oxygen vacancies and lattice oxygen for the adsorption/oxidation of Hg^0^ on the regenerated sample’s surface [27], enhancing Hg^0^ adsorption. Additionally, the diffraction peaks corresponding to Cu-BTC in the composite material completely disappeared in R-Cu-BTC@MWS, confirming that the composite material was fully decomposed during regeneration, resulting in the formation of the derived carbon material R-Cu-BTC@MWS.

The surface chemical characteristics of the samples before and after regeneration are shown in Figure 4b. It was found that absorption bands in the aliphatic CH vibrational region in the R-Cu-BTC@MWS sample formed after regeneration was significantly enhanced. This is due to the dissociation of the organic ligands during the formation of the derived carbon materials, accompanied by the shedding of oxygen from some of the oxygen-containing functional groups to free oxygen, which ultimately combined to form alkanes and escaped. In addition, compared with the composite material before regeneration, the intensity of the absorption bands corresponding to the vibrational region of the oxygen-containing functional groups in the regenerated sample was weakened, indicating a decrease in the number of these groups on the surface. This is partly because the mercury adsorption process consumed many functional groups, and the O_2_ introduced during regeneration could only replenish some of them. Moreover, the results from the weight loss characteristics in the previous section showed that the temperature caused the direct bond-breaking reaction of many functional groups.

#### 3.2.4. Microscopic Morphology

**Figure 5:**
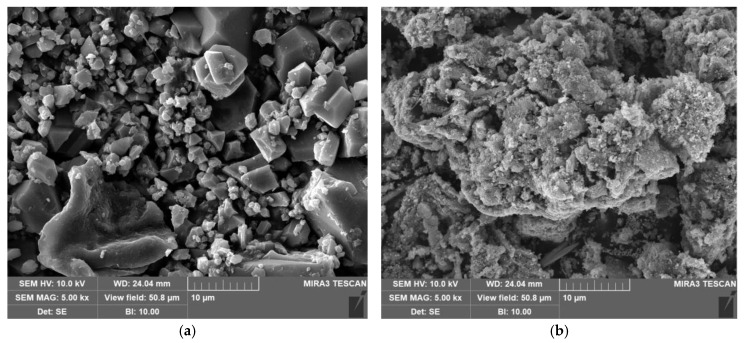
Microscopic morphology of the samples. (**a**) Cu-BTC@MWS. (**b**) R-Cu-BTC@MWS.

The micro-morphology of the samples before and after regeneration is shown in Figure 5. Among them, the Cu-BTC@MWS sample prepared by composite synthesis revealed that the modified biochar was surrounded by octahedral-shaped Cu-BTC material, with only part of the biochar edges exposed. whereas Cu-BTC solid particles could be observed on the surface of the regenerated samples after high-temperature heating, indicating that the Cu-BTC structure had decomposed during regeneration, resulting in the formation of the derived carbon material R-Cu-BTC@MWS. Additionally, the surface of the R-Cu-BTC@MWS sample was porous and interconnected, facilitating the diffusion and adsorption of Hg^0^, and it also showed more polymer clusters on the surface. This occurred mainly because, under an O_2_ atmosphere, the metal ions in the composite adsorbent underwent redox reactions, producing metal oxides and metal monomers. These metal oxides and monomers form on the surface, providing active sites for chemical adsorption of Hg^0^.

#### 3.2.5. Carbon Chain Structure Composition

**Figure 6:**
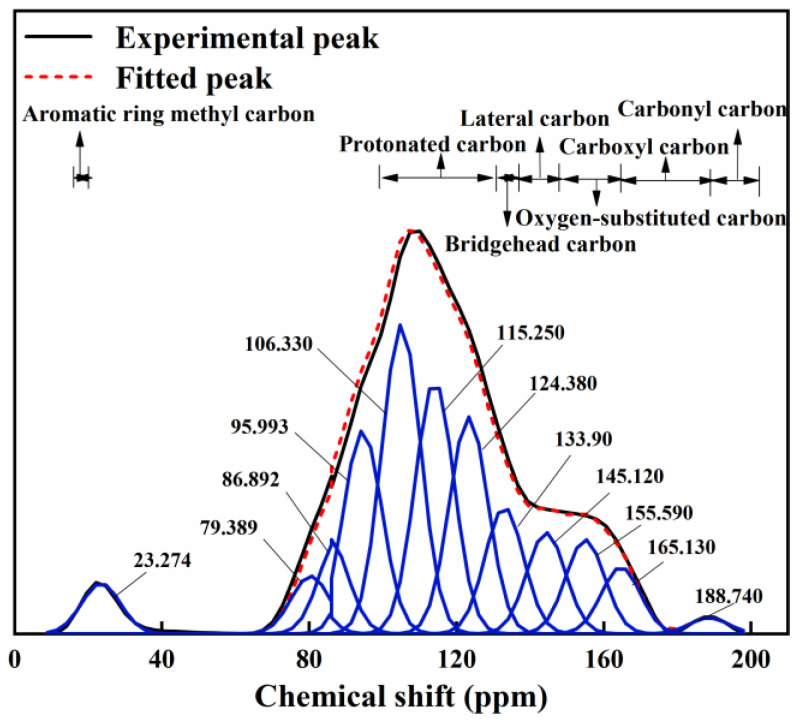
Peak fitting results of the ^13^C-NMR.

^13^C-NMR spectra were performed on the acid-washed, demagnetization-treated R-Cu-BTC@MWS samples. The resulting NMR patterns and fitted peak results are shown in Figure 6. Additionally, the fitted areas were normalized to indicate the relative content based on the chemical shift assignment for different carbon structures, with results shown in Table S2. The analysis reveals that the most prevalent carbon type is the bridgehead carbon, constituting 40.9%, followed by protonated aromatic carbon at 31.8% and side-branch aromatic carbon at 9.1%. The structural parameters of the regenerated R-Cu-BTC@MWS samples are calculated and listed in Table S3. According to the $X_{\mathrm{BP}}^{*}$ value of 1 in the calculated results, it can be seen that the overall molecular structure of the regenerated sample had strong ring tension, which corresponded to high reactivity, and its molecular structure was inferred to include two anthracene benzenes, two pyridine nitrogens and one furan, based on the calculable relative molecular mass.

### 3.3. Molecular Modeling of Regenerated Samples

**Figure 7:**
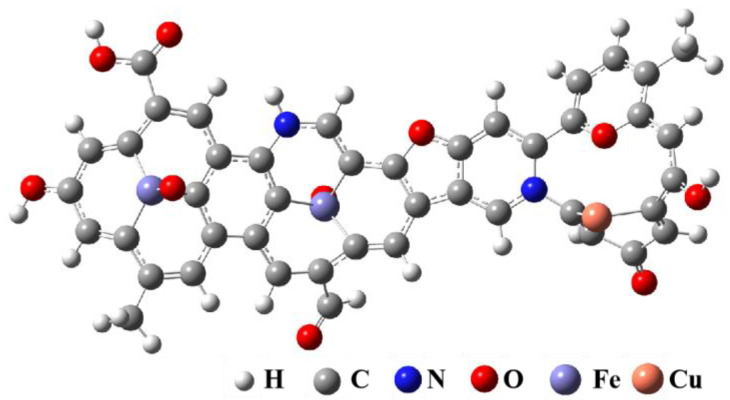
Optimized molecular structure modeling of R-Cu-BTC@MWS.

Based on the microscopic morphology and lattice characterization of the R-Cu-BTC@MWS regenerated samples, combined with the results of elemental analysis, surface functional groups, and carbon chain analysis, and based on preliminary determination of the molecular formula and carbon structure, the corresponding 2D molecular model was constructed by using ChemDraw Professional 23, and the infrared prediction spectrum of the corresponding model was calculated by using Chem3D Ultra 14 and compared with the experimental spectrum. By adjusting the connection position and connection mode of atoms several times, the predicted infrared spectra and the experimental infrared spectra were finally obtained to ensure the authenticity and reasonableness of the constructed configurations. The final molecular formula and relative molecular mass are C_44_H_25_O_10_N_2_Fe_2_Cu, *M*_r_ = 917. The corresponding molecular configuration and ^13^C-NMR spectrum are shown in Figure S5 and Figure S6, respectively, with a high degree of agreement, and the final optimized configuration is shown in Figure 7.

### 3.4. Adsorption and Regeneration Mechanism

#### 3.4.1. Determination of Adsorption Sites

**Figure 8:**
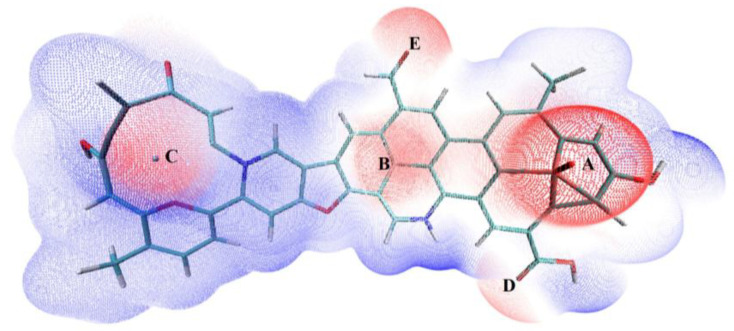
Molecular surface electrostatic potential of R-Cu-BTC@MWS.

The wave function and molecular surface electrostatic potential (ESP) of the regenerated samples of R-Cu-BTC@MWS were calculated using Multiwfn 3.8 and are shown in Figure 8. The sites with high electrostatic potential on the molecular surface of the derived carbon materials were dispersed around the metal oxides, Cu^0^, and oxygen-containing functional groups. Therefore, the metal oxides, Cu^0^, and oxygen-containing functional groups were predicted to be the main adsorption sites in the R-Cu-BTC@MWS samples. These adsorption sites were labeled as A, B, C, D, and E, respectively. A and B correspond to Fe_3_O_4_ metal oxides; C corresponds to Cu^0^; and D and E correspond to oxygen-containing functional groups, mainly including carboxyl and aldehyde groups. Compared with the composites before regeneration, the Cu^2+^ initially present in the samples was reduced to Cu^0^, which was co-distributed with the metal oxides and oxygen-containing functional groups formed during regeneration in the molecular structure of R-Cu-BTC@MWS. This Cu^0^ acted as a chemical adsorption site directly involved in Hg^0^ adsorption on the composite surfaces.

**Figure 9:**
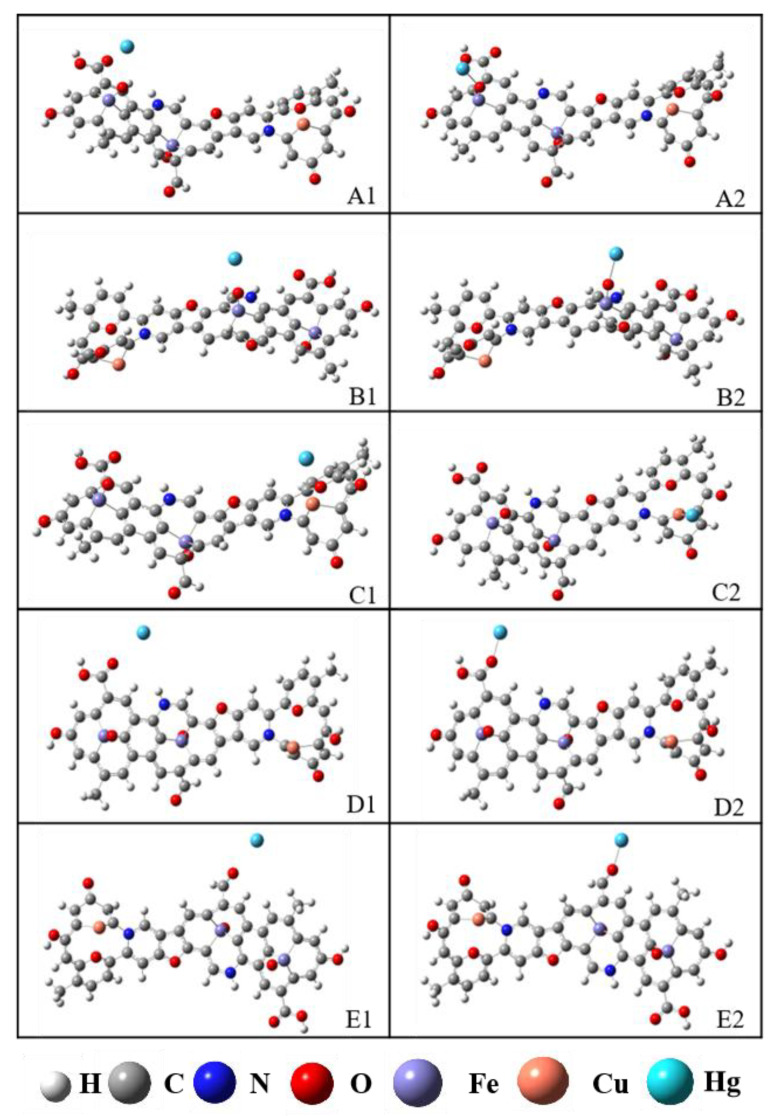
Adsorption configurations for the adsorption of Hg^0^ at different active sites on the surface of the R-Cu-BTC@MWS regeneration sample. (**A1**) Initial state at A site; (**A2**) Final state at A site; (**B1**) Initial state at B site; (**B2**) Final state at B site; (**C1**) Initial state at C site; (**C2**) Final state at C site; (**D1**) Initial state at D site; (**D2**) Final state at D site; (**E1**) Initial state at E site; (**E2**) Final state at E site.

To further investigate the reaction mechanism of Hg^0^ on the surface of the R-Cu-BTC@MWS, the adsorption process of Hg^0^ on the surface of the regenerated samples was theoretically simulated and calculated by placing it on the tops of A, B, C, D, and E in the molecular model of R-Cu-BTC@MWS, respectively. The configurations before and after adsorption are shown in Figure 9. A1–E1: X1 denotes the initial state where no reaction has occurred at this site, while X2 denotes the final state where adsorption at this site is complete. The adsorption energies and key lengths corresponding to the adsorption sites A, B, C, D, and E were calculated, and the results are shown in Table 2.

It was found that for the five adsorption sites in the regenerated samples, the absolute value of Hg^0^ adsorption energy exceeded 42 kJ/mol, indicating chemical adsorption. Among these, the adsorption energies at sites A and B were −182.45 kJ/mol and −171.36 kJ/mol, respectively. The highest absolute value of adsorption energy and the shortest key length corresponded to the best adsorption capacity. The adsorption energy at Cu^0^ site C was −156.83 kJ/mol, which was lower than that at the metal oxide sites (A and B). For the oxygen-containing functional group sites, the carboxylic acid site (D) showed weaker Hg^0^ adsorption than the aldehyde site (E), with adsorption energies of −98.71 kJ/mol and −122.08 kJ/mol, respectively. Therefore, metal oxides Fe_3_O_4_ and Cu^0^ had stronger Hg^0^ adsorption capacities than oxygen-containing functional groups.

During the specific adsorption reaction, the atomic valence state of the introduced Fe^3+^ changed because Fe, as a high-valent metal, helped promote Hg^0^ adsorption by providing empty orbitals to accept the escaping electrons. However, the overall electric field distribution within the molecular structure of the composite adsorbent was modified to enhance Hg^0^ adsorption by working synergistically with N [28]. In contrast, the adsorption sites A and B, associated with Fe_3_O_4_ in the regenerated sample R-Cu-BTC@MWS, mainly participated in Hg^0^ adsorption through the redox reaction of the metal oxide Fe_3_O_4_. Hg^0^ was directly oxidized to Hg^2+^, ultimately forming HgO.

For the Cu^0^ adsorption site C, the adsorption process of R-Cu-BTC@MWS on Hg^0^ mainly depended on the free O^2−^ on the surface of R-Cu-BTC@MWS and the lattice oxygen and oxygen vacancies in the oxygen-storage solid solution for its redox reactions with Hg^0^. Ultimately, the formation of complexes completed the adsorption of Hg^0^. Compared with the composite material before regeneration, this site did not rely on its high unsaturated coordination to achieve adsorption. Additionally, the Fe_5_CuO_8_ oxygen-storing solid solution present in the regenerated sample can also oxidize Hg^0^ that has diffused to the surface by supplying lattice oxygen and oxygen vacancies, thereby facilitating the removal of Hg^0^. For the oxygen-containing functional groups at adsorption sites D and E, since the oxygen in these groups had high electronegativity and strong electron-attracting ability, they could overlap with the Hg^0^ electron cloud through Lewis acid–base reactions when Hg^0^ was nearby, enabling Hg^0^ adsorption [29]. Therefore, compared to the oxygen-containing functional groups in the composites, which indirectly facilitated Hg^0^ adsorption on Cu^2+^, the surface functional groups of the regenerated R-Cu-BTC@MWS samples could directly interact with Hg^0^.

#### 3.4.2. Reaction Mechanisms

**Figure 10:**
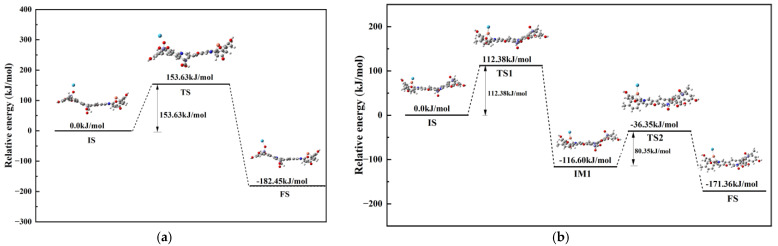
Adsorption reaction pathways for Hg^0^. (**a**) Reaction site A. (**b**) Reaction site C.

To better understand the adsorption reaction paths and mechanisms of Hg^0^ on the regenerated samples R-Cu-BTC@MWS, the adsorption sites A and C, which correspond to the metal oxides (Fe_3_O_4_) and Cu^0^ with the best adsorption effects, were examined separately. Simulations were performed by placing Hg^0^ at the top sites of A and C. The two reaction pathways are shown in Figure 10. At the adsorption site A, the primary process involved a redox reaction between Fe_3_O_4_ and Hg^0^, resulting in the formation of HgO on the surface of the regenerated sample. The steps of this adsorption process are as follows: (1) During calculations, Hg^0^ was placed about 3 Å from the reaction site, a distance sufficient to ensure initial interaction energies were negligible, establishing this as the zero-reference point for calculating adsorption energies. In the early reaction stage, Hg^0^ gradually moved closer to the sample surface, with the distance between O and Hg decreasing from 2.95 Å to 2.23 Å. The electron cloud of Hg^0^ influenced the Fe-O electrons, shifting them toward Hg^0^, and causing the Fe–O bond length to stretch from 1.52 Å to 1.59 Å. (2) As the electron cloud shifted, the oxidizing ability of the unsaturated oxygen-coordinated Fe^3+^ ions increased, weakly oxidizing Hg^0^ to Hg^+^, which also partially reduced Fe^3+^ to Fe^2+^. Concurrently, lattice oxygen was converted to free O^2−^ and released to the surface of the sample. Subsequently, Hg^+^ was further oxidized to Hg^2+^, elongating the Fe–O bond to 1.62 Å. The free O^2−^ then combined with Hg^2+^ to form HgO, with an O–Hg distance of 2.18 Å. The overall energy barrier for this process was 153.63 kJ/mol.

As for the adsorption site C, it is known from the previous text that the adsorption of lattice oxygen, oxygen vacancies, and unsaturated metal Cu^+^ on Hg^0^ mainly occurred, and Hg^0^ eventually formed a complex with Cu^+^, existing on the surface of the regenerated samples. The adsorption reaction was mainly divided into three steps: (1) During calculations, Hg^0^ was placed about 3 Å from the reaction site, a distance sufficient to ensure initial interaction energies were negligible, establishing this as the zero-reference point for calculating adsorption energies. At this time, the distances between O and Hg and O and Cu were stretched from the original 2.98 Å and 2.80 Å to 3.82 Å and 2.99 Å, respectively, and the angle between O, Cu, and Hg was 78.92°, with strong repulsion between the electrons. The step corresponded to the activation energy barrier of 112.38 kJ/mol. (2) When Fe_3_O_4_ reacted with Hg^0^, part of the escaped O^2−^ and lattice oxygen in the oxygen-storing solid solution oxidized Cu^0^ to Cu^2+^ and formed oxygen vacancies, promoting the attraction of Cu^2+^ to Hg^0^. Cu^2+^ provided empty orbitals to accept the electrons that escaped from Hg^0^, and Hg^0^ was oxidized to Hg^+^, where Cu^2+^ was reduced to Cu^+^ to form [Cu^+^–Hg^+^]. At this time, the distances between O and Cu and Cu and Hg were 2.72 Å and 2.53 Å, respectively, and the angle between O, Cu, and Hg was 102.08°. As the bond angle increased, the interatomic electron repulsion weakened, and the activation energy barrier for this step was 80.35 kJ/mol. (3) As the reaction proceeded, Hg^+^ escaped to the surface of the free state O^2−^, was continuously oxidized to Hg^2+^, and under the electron repulsion, formed a stable [Cu^+^–Hg^2+^] coordination bond with Cu^+^ to complete the adsorption of Hg^0^. The final distance between Cu and Hg was 2.58 Å and the bond angle between O, Cu, and Hg was 135.12°.

**Figure 11:**
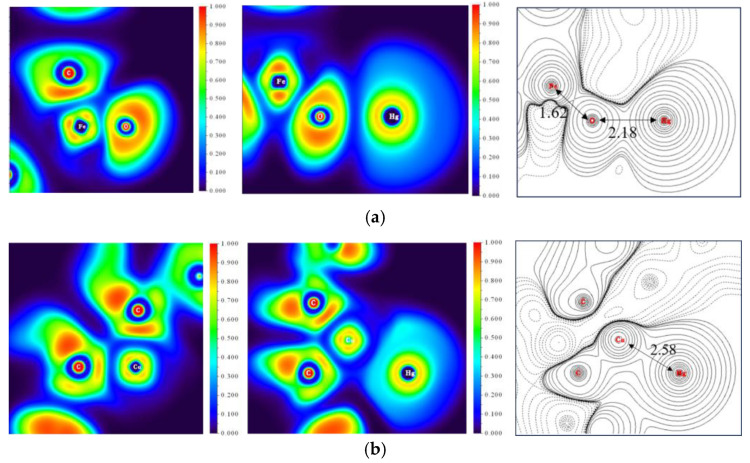
Potential distribution and differential charge density. (**a**) Reaction site A; (**b**) Reaction site C.

Combined with the results of the reaction path investigation obtained in the previous sections, it can be seen that the electron transfer occurred in the adsorption process of R-Cu-BTC@MWS during the adsorption of Hg^0^. Therefore, the corresponding electron transfer process was studied by analyzing the ELF potential distribution and differential charge density, and the results corresponding to adsorption sites A and C are shown in Figure 11. The red color in the figure represents the high electron density in the region, indicating that covalent bonds may be formed in the region, and the blue color suggests that the electron density in the region was lower. Ionic bonds or no bonds may be formed in the region [30].

Among them, for the adsorption site A, the electron density distribution of Fe atoms and O atoms changed significantly before and after the reaction. After the reaction, the electron density of Fe atoms was concentrated in the vicinity of O atoms, while the electron density between Fe and O atoms was reduced. The ionic bonding force formed was weakened, corresponding to the larger Fe–O bonding length, which was conducive to the next step of oxygen atom adsorption by Hg^0^. Meanwhile, the electron density distribution of O atoms was expanded, because during the redox reaction, iron oxide, as an oxidizing agent, accepted the electrons from the reductant Hg^0^, thereby oxidizing Hg^0^ to Hg^+^. The O^2−^ escaped to the surface of the regenerated sample, further oxidized Hg^+^ to Hg^2+^, and formed HgO. Combined with the results of the differential charge densities, the electron densities of the Fe, O, and Hg atoms were increased, and the positions of the Hg atoms and O atoms were located in the same electron domain. The range of the formed electron domain was larger than that formed by the Fe atoms and O atoms, proving further evidence that Hg^2+^ bonded with O^2−^ to form HgO. The corresponding Fe–O bond length was smaller than the Hg–O bond length. During the entire reaction bonding process, Fe_3_O_4_ directly acted as an oxidant to oxidize Hg^0^, which served as the primary adsorption site for Hg^0^ adsorption in the regenerated sample.

For the adsorption site C, the electron distribution of Cu atoms changed considerably before and after the reaction, and the central electrons of the Cu atoms increased because Cu^0^ underwent a redox process, resulting in a rearrangement of the inner electronic structure. Cu^+^ was in a more stable state after the reaction. During the process of Hg^0^ being close to the adsorbent, part of the electrons of the C atom were enriched around the Cu atom. At the same time, under the action of O^2−^ on the surface and the lattice oxygen in the oxygen-storing solid solution, Cu^0^ was oxidized to Cu^2+^, strengthening the attraction to the electron cloud of the Hg atom. Cu^2+^ provided empty orbitals to accept electrons escaping from Hg^0^ with decreasing valence. Hg^+^ was oxidized by free O^2−^ to form Hg^2+^, and eventually formed a coordination bond with Cu^+^. Similar to HgO, the Cu atoms were in the same electron domain as the Hg atoms in the differential charge density diagram, further indicating the formation of Cu–Hg bonds.

**Figure 12:**
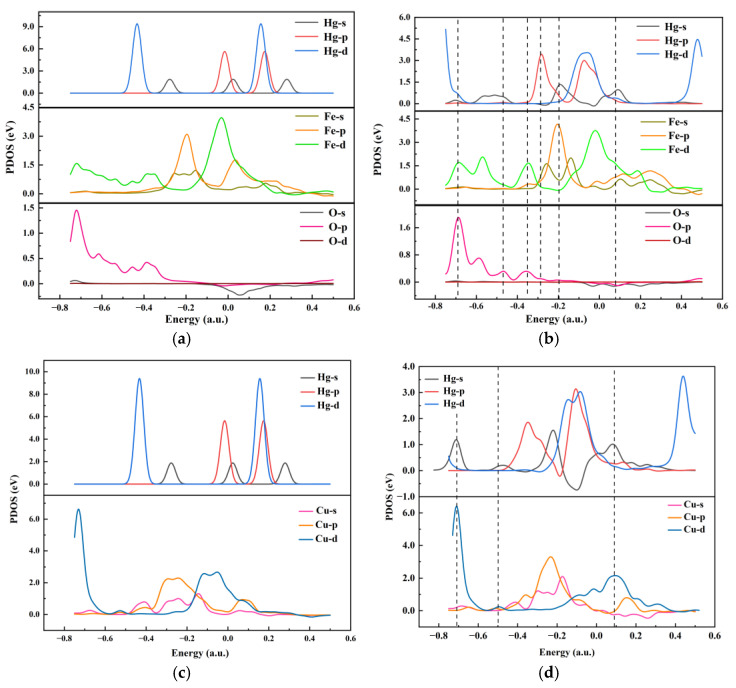
Projected density of states. (**a**) Adsorption site A before adsorption of Hg^0^, (**b**) adsorption site A after adsorption of Hg^0^, (**c**) adsorption site C before adsorption of Hg^0^, and (**d**) adsorption site C after adsorption of Hg^0^.

In addition, the projected density of states during the reaction was calculated based on DFT to analyze the degree of contribution of different atomic orbitals to the bonding during the Hg^0^ adsorption reaction. The bonding mechanism of the composite adsorbent to Hg^0^ was investigated from the perspective of electronic orbitals, and the results corresponding to the adsorption sites A and C are shown in Figure 12. For adsorption site A, the corresponding *s*, *p*, and *d* orbitals of Hg atoms jumped from higher to lower energy levels before and after the reaction, indicating that the adsorbed product was more stable [31]. Hg-*s* orbitals decreased in energy level and overlapped with O-*p* orbitals at −0.48 (a.u.) and 0.08 (a.u.), while Hg-*p* orbitals overlapped with O-*p* at −0.28 (a.u.). The adsorption process of the regenerated sample promoted the activation of the outer electrons of the Hg atoms and electron leaps, leading to bonding with the O atoms. During this process, the Fe-*d* orbitals underwent energy level cleavage and orbital overlap with O-*p* at −0.35 (a.u.) and −0.59 (a.u.), suggesting that the outer electrons of the Fe atoms were electronically rearranged and re-bonded with the O atoms during the reaction process.

Similar to the A adsorption site, for the adsorption site C, the corresponding *s*, *p*, and *d* orbitals of the Hg atoms after the reaction were moved to lower energy levels to react with Cu^+^ to produce more stable complexes. The orbital overlap of Hg-*s* and Cu-*d* orbitals in this process occurred at −0.71 (a.u.), −0.50 (a.u.), and 0.09 (a.u.), thus verifying the conclusions from the previous study.

## 4. Regeneration Mechanism

**Figure 13:**
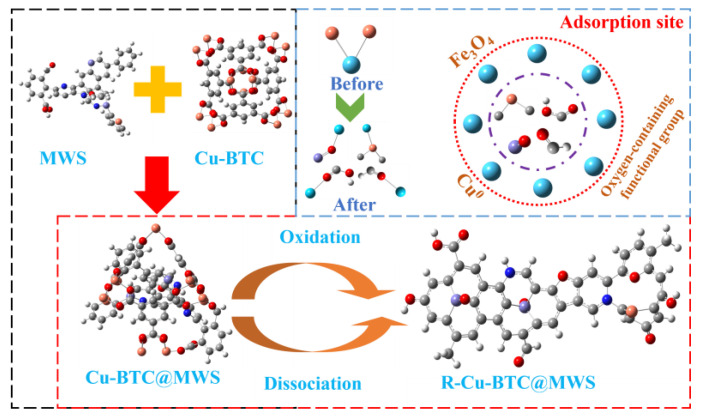
Regeneration mechanism of R-Cu-BTC@MWS.

Combining the analysis results above, the regeneration mechanism of the Cu-BTC-based modified biochar composite adsorbent is shown in Figure 13. Before regeneration, the composite was based on Cu-BTC and MWS, while the modified biochar loaded with Cu-BTC enhanced Hg^0^ adsorption on its surface through metal synergism. This process altered the electric field distribution, promoted valence changes, and provided carbon frameworks and oxygen-containing functional groups. Ultimately, Hg^0^ diffused to the surface and reacted with and bonded to the exposed unsaturated Cu^2+^ sites on Cu-BTC, thereby achieving Hg^0^ removal. During regeneration, the carbon component oxidized, and organic ligands disintegrated, causing the overall structure of the Cu-BTC-based modified biochar to collapse and form the derived carbon material R-Cu-BTC@MWS. The metal oxides, singlet copper, and oxygen-containing functional groups on the surface of the regenerated samples serve as adsorption sites, directly engaging in redox reactions with Hg^0^. Additionally, the oxygen-storing solid solution supplies lattice oxygen and vacancies that facilitate these reactions.

## 5. Industrial Application

**Figure 14:**
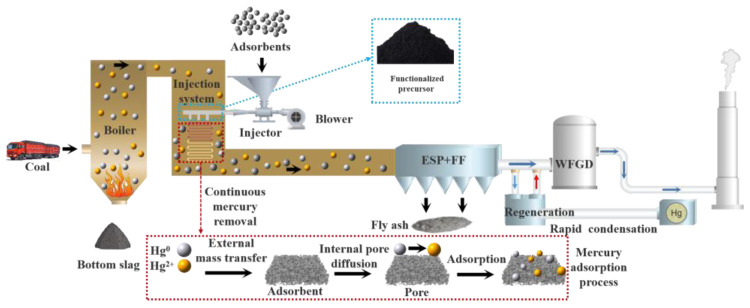
Background illustration of an industrial application.

The industrial application background is illustrated in Figure 14. By leveraging the high-temperature environment provided by flue gas during coal combustion in power plant boilers, the synthesized functionalized precursor materials are calcined. This process ingeniously integrates the utilization of flue gas waste heat with the synthesis of composite materials, enabling simultaneous control of the material’s pore structure and high dispersion of active sites during the high-temperature calcination stage. In this process, the prepared composite materials, with their hierarchical porous characteristics and surface chemical modification effects, achieve efficient removal of Hg^0^ within a lower suitable temperature range as the flue gas flows. The dynamic synergistic removal mechanism formed by the coordination of the unsaturated metal sites of the MOF framework and the oxygen-containing functional groups of the biochar plays a key role. Additionally, based on the regenerative repair function of the composite material’s active adsorption sites, it can overcome the technical bottleneck of high energy consumption in traditional adsorbent separation, further achieving efficient separation and recycling of adsorbents.

## 6. Conclusions

- Temperature and O_2_ volume significantly affect the regeneration process. For R-Cu-BTC@MWS, the ideal regeneration conditions were 350 °C and an atmosphere of 5% O_2_ + 95% N_2_. Under these conditions, the maximum regeneration efficiency reached 92% of the fresh sample’s adsorbed capacity. The regeneration cycle efficiency showed a gradual decline as the number of cycles increased.
- Under optimal regeneration conditions, the overall structure of the Cu-BTC-based modified biochar composite adsorbent samples collapsed and was accompanied by the formation of the derived carbon material R-Cu-BTC@MWS. The molecular structure of the regenerated samples included two anthracene-benzenes, two pyridinium nitriles, and one furan.
- During the adsorption of Hg^0^ on the surface of R-Cu-BTC@MWS, Fe_3_O_4_, as the main adsorption site, was directly involved in the redox reaction of Hg^0^ to realize the mercury removal, and the corresponding adsorption energy value of the adsorption configuration was the largest; the Cu^0^ needed to rely on the O^2−^ that escaped to the surface of R-Cu-BTC@MWS and the lattice oxygen and oxygen vacancies in the oxygen-stored solid solution to carry out the redox of Hg^0^ and itself and finally complete the removal process.

## Figures and Tables

**Table 1:** Ultimate analysis of the samples.

Samples	Ultimate Analysis/%						Atomic Ratio	
	C	H	O	Fe	Cu	N	H/C	O/C
Cu-BTC@MWS	37.3	0.9	21.7	1.1	4.4	34.6	0.02	0.58
R-Cu-BTC@MWS	57.6	2.7	17.4	12.3	6.9	3.1	0.05	0.30

**Table 2:** Adsorption parameters of Hg^0^ on the surface of the R-Cu-BTC@MWS regeneration sample under different adsorption configurations.

Adsorption Configurations	Adsorption Energy (kJ/mol)	Key Length (Å)
A	−182.45	2.18
B	−171.36	2.20
C	−156.83	2.58
D	−98.71	2.32
E	−122.08	2.23

## Data Availability

The original contributions presented in this study are included in the article. Further inquiries can be directed to the corresponding author.

## Supplementary Materials

The following supporting information can be downloaded at: https://www.mdpi.com/article/10.3390/polym17182474/s1, Figure S1: The total amount of mercury adsorbed per unit mass of biochar; Figure S2: The mercury adsorption penetration coefficient of biochar; Figure S3: Flowchart of the sample’s preparation process; Figure S4: Hg^0^ removal characteristics of samples; Figure S5: The results of the comparative experiment; Figure S6: Molecular structure modeling of the R-Cu-BTC@MWS sample; Figure S7: Comparison of ^13^C-NMR predicted calculated spectra with experimental spectra; Figure S8: Absorption and desorption curves and pore size distribution of the materials; Figure S9: Molecular surface electrostatic potential of Cu-BTC@MWS; Figure S10: Adsorption configurations for the adsorption of Hg^0^ at different active sites on the surface of Cu-BTC@MWS; Table S1: Mass fraction of biomass and mercury adsorption penetration coefficients of biochars within the scope of different particle sizes; Table S2: Carbon structure attribution and fitting parameters; Table S3: Molecular structure parameters; Table S4: Pore structure parameters of samples; Table S5: Adsorption parameters of Hg^0^ on the surface of Cu-BTC@MWS under different configurations.
