# Supplementary material for: Study on the Regeneration-Cycle Mechanism of Cu-BTC@MWS Composites Following Mercury Adsorption
Source: Polymers (Basel). 2025 Sep 12;17(18):2474. doi: 10.3390/polym17182474 (PMC12473158; doi:10.3390/polym17182474)
Supplement: Supplementary file 1 [file polymers-17-02474-s001.zip › polymers-3828991-supplementary.pdf]

Supplementary Materials

Study on the Regeneration-Cycle Mechanism of Cu-BTC@MWS Composites Following Mercury Adsorption

List of Supplementary Materials

S1. The selection of walnut shells and particle size

S2. Preparation of Samples

S3. Fixed-bed mercury removal experimental system

S4. Carbon structure attribution and fitting parameters

S5. Pore structure

S6. The results of mercury adsorption energies on Cu-BTC@MWS

S1. The selection of walnut shells and particle size

We have explained why walnut shells were selected as the research object and commented on the selection of particle size in the original text as follows:

(1) The selection of walnut shells as the research object: The average mercury content of China's coal is 0.15 mg/kg, while Shanxi Province has abundant coal resources, a huge number of coal-fired power plants, and a variety of coal types. The mercury content in Shanxi coal is higher than the national average of 0.22 mg/kg. China has required the mercury concentration limit in the flue gas of coal-fired power plants to be 30 μg/m^3 since January 1, 2015. The biomass resources in Shanxi Province are relatively abundant, including walnut shells, corn cobs, and cotton stalks, among which the walnut production ranks second in the country. The utilization of biomass resources was undoubtedly a green and low-carbon effort; the exploration of low-cost coking processes and adsorption processes was a necessary prerequisite for the use of biochar.

We selected walnut shells (WS), corn cobs (CC), cotton stalks (CS) and coconut shells (CH) as raw materials and completed the preparation of different kinds of biochar samples under the same conditions (pyrolysis temperature was 600°C, and pyrolysis time was 10 min). The mercury adsorption characteristics of biochar samples are shown in Figs. S1 and S2. During the 200 min adsorption time, the mercury adsorption performance of WS was the strongest; the initial penetration rate and the final penetration rate were the lowest, which were 6.67% and 66.67%, respectively. In addition, the total amount of mercury adsorbed per unit mass (q) of WS was 2029 ng/g, CH was 724 ng/g, CC was 767 ng/g, and CS was 1102 ng/g. Based on these results, walnut shells were selected as the raw material for the study.

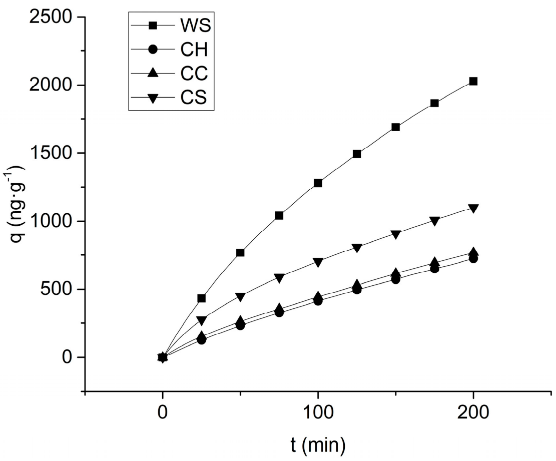

Figure S1. The total amount of mercury adsorbed per unit mass of biochar

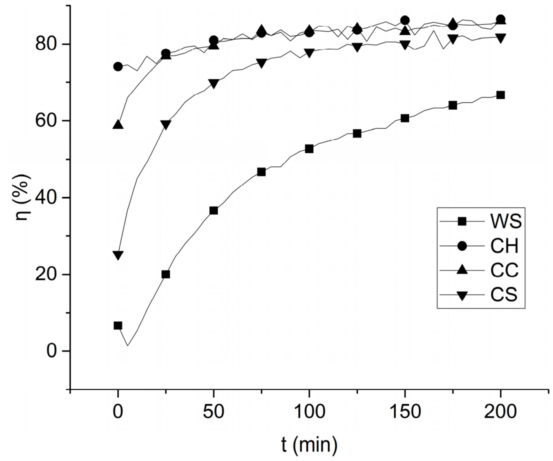

Figure S2. The mercury adsorption penetration coefficient of biochar

(2) The selection of particle size: However, few studies on the effects of different particle sizes on the mercury adsorption characteristics of sorbents have been reported. These few studies have been focused on fly ash as the research object. For instance, Huang[S1] considered three different particle size ranges of fly ash and found that the size of fly ash had an impact on the mercury transformation, and the conversion ratio of Hg^{2+} to total Hg increased with increasing size of fly ash. However, the pore structures of fly ash with different particle sizes were not analyzed, and fewer particle size ranges were selected (only three ranges).

In order to obtain the most suitable size range for the study, biomass and the corresponding prepared biochar for eight particle size ranges were studied. The walnut shell materials were all from the same production area. In the process of calculating the mass fractions of different particle sizes of biomass, the 200 g sample was obtained by the quartering method from a 1 kg walnut shell sample, and then the particle size classification was performed using a vibrating machine to obtain samples with eight different particle size ranges. These ranges were >425 μm, 270 μm to 425 μm (50 mesh), 150 μm to 270 μm (100 mesh), 106 μm to 150 μm (150 mesh), 75 μm to 106 μm (200 mesh), 58 μm to 75 μm (250 mesh), 48 μm to 58 μm (300 mesh), and < 48 μm. The weights of samples were calculated by electronic balance, in order to obtain the mass fractions of the biomass samples within different particle size ranges. The biomass

samples within different particle size ranges were pyrolyzed at 600°C for 10 min. The measured mercury adsorption penetration coefficients (η) of biochar (120 min adsorption time) were determined by average value obtained through three parallel experiments.

The results are shown in Table S1. The four particle size ranges, 150 μm to 270 μm (100 mesh), 106 μm to 150 μm (150 mesh), 75 μm to 106 μm (200 mesh), and 58 μm to 75 μm (250 mesh), accounted for a mass fraction of 86.44%, and the mercury adsorption characteristics of the corresponding biochar were better. In addition, although the mercury adsorption effects in the particle size ranges of 48-58 μm and <48 μm were relatively higher, the improvement effect was not obvious, and the mass fractions were too low, resulting in a lack of practical application economic value due to the high grinding cost required.

In summary, the particle size of 58 μm to 75 μm was selected for study, based on not only the mass distribution but also the corresponding mercury adsorption characteristics and its economy.

Table S1. Mass fraction of biomass and mercury adsorption penetration coefficients of biochars within the scope of different particle sizes

Item	Particle size (μm)							
	>425	270-425	150-270	106-150	75-106	58-75	48-58	< 48
Mass fractions of biomass (%)	3.91	3.49	31.99	24.93	15.76	13.76	2.55	3.61
Mercury adsorption penetration coefficients (η) of biochars (%)	95.66	92.77	84.77	67.33	59.33	56.67	56.43	56.42

[S1] Huang, H.; Luo, J. Effect of various fly ash compositions on mercury speciation transformation. *J. Proceeding of the Cess.* **2010**, *30*:70-75.

S2. Preparation of Samples

Walnut shell biochar (MWS) modified by 10%Fe-2%Cu was prepared by a sol–gel combined with the co-precipitation method. Walnut shells were ground and sieved to obtain walnut shell biomass with a particle size of 250 mesh (58–75 μm), a weight of 15 g biomass, 8.643 g $FeCl_3 \cdot 6H_2O$ and 1.403 g $CuSO_4 \cdot 5H_2O$. The mass of the metal compound is obtained from Equation (S1):

$$m_{\text{compound}} = \frac{15}{0.84} \times A\% \times \frac{M_{\text{compound}}}{M_{\text{A}}} \tag{S1}$$

where m_{compound} is the compound mass of required element A, $A\%$ is the doping mass ratio of element A and M_{A} and M_{compound} are the molar masses of element A and the compound of A, respectively.

The above biomass and metal compounds were dissolved in a mixed solution of 100 mL anhydrous ethanol and 20 mL deionized water and stirred well. Then, 15 mL 1,2-epichlorohydrin and 1 mL DMF were added to form a sol, and after heating in a water bath at 40 °C for 24 h, 2.8 mL ethyl orthosilicate mixed with 0.7 mL anhydrous ethanol was added to the sol to disperse the sol as a wet gel. The precursor material (FeCu/precursor) was obtained by heating it in a water bath at 60 °C for 24 h and then drying and grinding at 70 °C. FeCu/precursor (6–7 g) was weighed and heated at 800 °C for 10 min under N_2 airflow with a flow rate of 200 mL/min to obtain MWS.

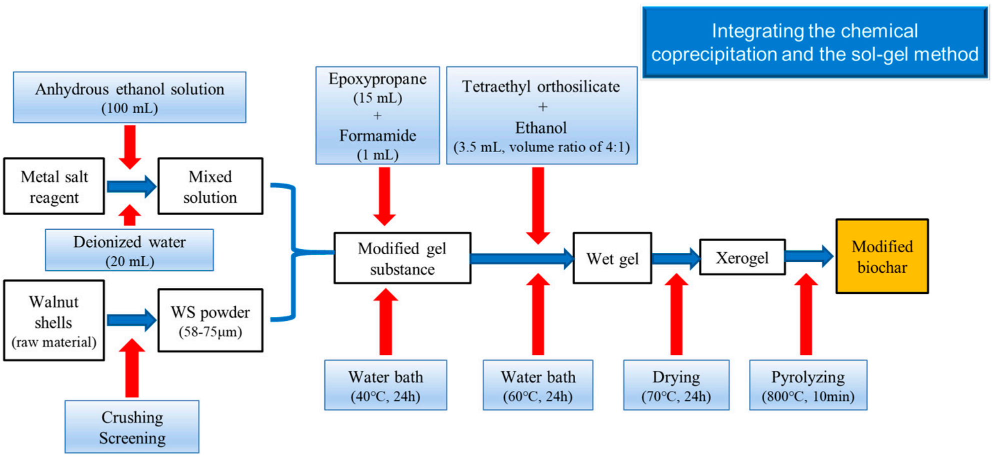

Figure S3 Flowchart of the sample's preparation process

The MOFs material Cu-BTC samples were mainly obtained by the in situ growth method, in which first 3.0 g of 1,3,5 homophthalic acid ($C_9H_6O_6$) was firstly dissolved in a mixed solution of 30 mL of N,N-dimethylformamide and 60 mL of anhydrous ethanol, and 6.0 g of copper nitrate trihydrate ($Cu(NO_3)_2 \cdot 3H_2O$) was weighed to be dissolved in 60 mL of deionized water; the two solutions were then mixed and stirred with a magnetic stirrer, and then the mixed solution was clarified and then moved to a stainless steel hydrothermal synthesis reactor with PTFE lining. The crystallization reaction was carried out at a constant temperature (85 °C) for 20 h. After that, it was taken out and left to cool down to room temperature. Finally, centrifugation was performed and the blue crystals obtained after several washes and drying were noted as Cu-BTC samples.

Cu-BTC@MWS adsorbents were prepared by in situ growth method based on the structural design of modified biochar doped with Fe/Cu polymetallic (MWS) and Cu-BTC as MOF materials, both of which contain unsaturated metal centers and oxygen-containing functional groups. Firstly, the corresponding modified biochar, homobenzoic acid and copper nitrate trihydrate were weighed according to the loading ratio conditions and dissolved in a mixed solution consisting of N,N-dimethylformamide, deionized water and anhydrous ethanol, and then loaded into a reactor with magnetic stirring for 1 h. The reaction was carried out at a constant temperature (85 °C) for 20 h. The supernatant was poured after standing and cooling and the residual solids were washed with anhydrous ethanol for 2-3 times, and then the products obtained were then extracted by using a vacuum drying oven and then washed by using a vacuum drying oven. The obtained product was dried at 100 °C for 12 h in a vacuum drying oven, and the composite adsorbent sample was finally obtained. The samples were labeled as Cu-BTC@MWS (50%) according to the loading ratio of MWS.

To investigate the influence of MWS loading ratios, this work examined the mercury removal characteristics of composite adsorbents with MWS loading ratios of 5%, 10%, 15%, 30%, 50%, 60%, and 70%. These were compared with the removal performance of MWS and Cu-BTC alone, with results presented in Figure S4. The

study revealed that Cu-BTC material exhibited superior mercury removal performance compared to modified biochar. For Cu-BCT@MWS, the mercury adsorption capacity of the composite samples demonstrated an overall trend of initially increasing and then decreasing as the loading ratio of the MWS increased. The optimal loading ratio is 50%, yielding a mercury adsorption capacity of 239.18 μg/g. Compared to the MWS and Cu-BTC samples, the corresponding removal efficiencies increased by 71.0% and 61.3%, respectively.

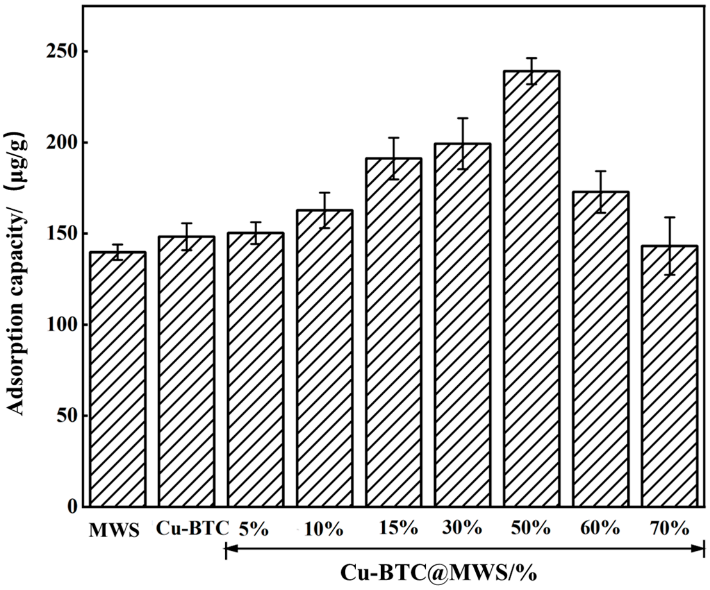

Figure S4. Hg^0 removal characteristics of samples

S3. Fixed-bed mercury removal experimental system

A fixed-bed mercury removal experimental system was constructed to determine the mercury removal performance of the samples, which consisted of a gas distribution device, a mercury generator, a fixed-bed reaction device, a mercury detection device and a tail gas treatment device. The gas distribution device mainly included gas cylinders and mass flow meters, in which N_2 was used as the carrier gas to carry Hg^0 into the reaction system and acted as the balance gas to regulate the total gas flow in the experimental system. The flow rate of the carrier gas was set to 500 mL/min, and to satisfy the requirement of the VM3000 mercuric meter on the inlet volume, the flow rate of the balance gas was set to 900 mL/min.

The mercury generator consisted of a mercury permeation tube, a water bath and a U-shaped glass tube. The mercury permeation tube inside the U-shaped glass tube was heated by the heat generated from the water bath to produce the Hg^0 required for the experiment, and the temperature of the water bath was adjusted to control the experimental concentration of Hg^0. In this study, the initial Hg^0 concentration was set to 100 ± 5 μg/m^3. The fixed-bed reaction device consisted of a tube furnace, a quartz tube (inner diameter Φ16.5 mm) and a temperature control device, in which the quartz tube served as the reaction site of the sample. The quartz boat was equipped with a porous quartz sieve plate as the carrying platform for the samples, whereas the temperature control device was used to investigate the variation rule of sample mercury removal performance with temperature by adjusting the temperature of the tube furnace. The tail gas device was mainly composed of KI-modified coconut shell activated carbon, which was used to treat the unadsorbed mercury during the reaction process. Before the adsorption experiments, the water bath was opened, the carrier gas was introduced, and the fixed bed temperature was set as the adsorption temperature. Then, the carrier gas and the equilibrium gas were introduced into the VM3000, and the adsorbent was placed into the quartz tube reactor to start the adsorption experiments after the inlet mercury concentration stabilized at 100 ± 5 μg/m^3.

This paper focused on the adsorption characteristics of Hg^0, so to eliminate the corresponding interference in the experimental process, before the experimental gas entered the VM3000, the $SnCl_2$ solution was used to reduce the Hg^{2+} (some Hg^0 oxidized on the surface of samples and escaped) in the experimental gas to Hg^0, and then into the VM3000 with the original Hg^0 in the flue gas, thus the adsorption characteristics on Hg^0 were obtained. As a result, the sorbent performance parameters are not exaggerated.

Moreover, combining with the results obtained by TPD, it is found that there is some elemental mercury oxidized on the surface of the samples. In order to verify the oxidized elemental mercury on the surface of samples that escaped, a comparative experiment was performed (using $SnCl_2$ solution and not using $SnCl_2$ solution before the flue gas entered the VM3000) under the same adsorption conditions. The results are shown in Figure S5. The values detected with the former were more than with the latter. Therefore, this mercury may have escaped during the adsorption process.

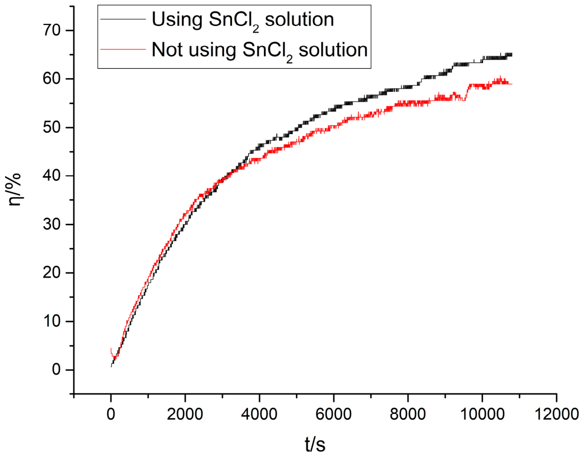

Figure S5. The results of the comparative experiment

Additionally, this elemental mercury (oxidized on the surface of the samples and escaped) was as the outlet mercury in the mercury balance calculation.

S4. Carbon structure attribution and fitting parameters

Table S2. Carbon structure attribution and fitting parameters

Carbon structure	Structural attribution	Chemical shift (ppm)	Structural ratio/% R-Cu-BTC@MWS
Qiaotou Aromatic Carbon	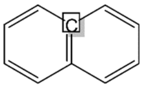	129-137	40.9
Protonated Aromatic Carbon	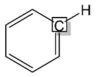	100-129	31.8
Side Branch Aromatic Carbon	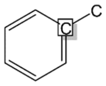	137-148	9.1
Oxygen-Substituted Aromatic Carbons	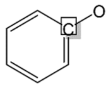	148-165	9.1
Aromatic Ring Methyl Carbons	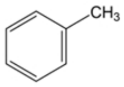	16-22	4.5
Carboxylic Carbon	—COOH	165-188	2.3
Carbonyl Carbon of Aldehyde	—CHO	188-205	2.3

Table S3. Molecular structure parameters

Samples	f_a [1]	f_a' [2]	f_a^N [3]	f_a^H [4]	f_a^C [5]	f_a^B [6]	f_a^S [7]	X_{BP}^* [8]
Cu-BTC@MWS	81.3	80	36	44	1.3	5.3	30.7	0.07
R-Cu-BTC@MWS	84.1	81.8	50	31.8	2.3	40.9	9.1	1

[1] Total aromatic carbon rate; [2] Aromatic ring carbon rate; [3] Non-protonated aromatic carbon rate; [4] Carbonyl carbon rate; [5] Protonated aromatic carbon rate; [6] Bridged aromatic carbon rate; [7] Lateral aromatic carbon rate; [8] Ratio of bridge carbon to perimeter carbon.

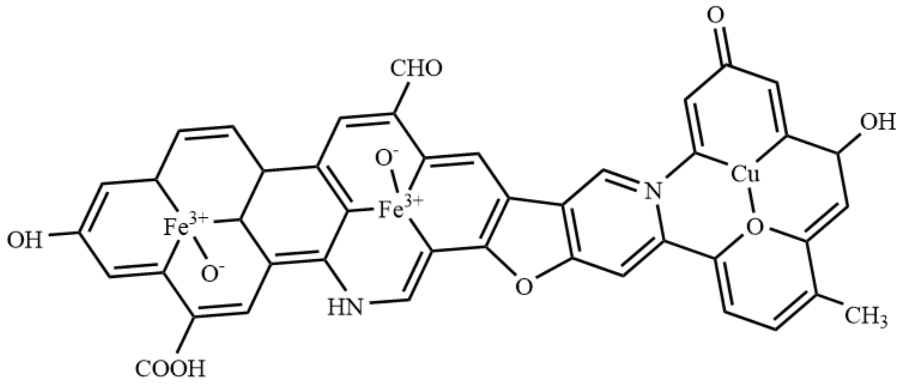

Figure S6. Molecular structure modeling of the R-Cu-BTC@MWS sample.

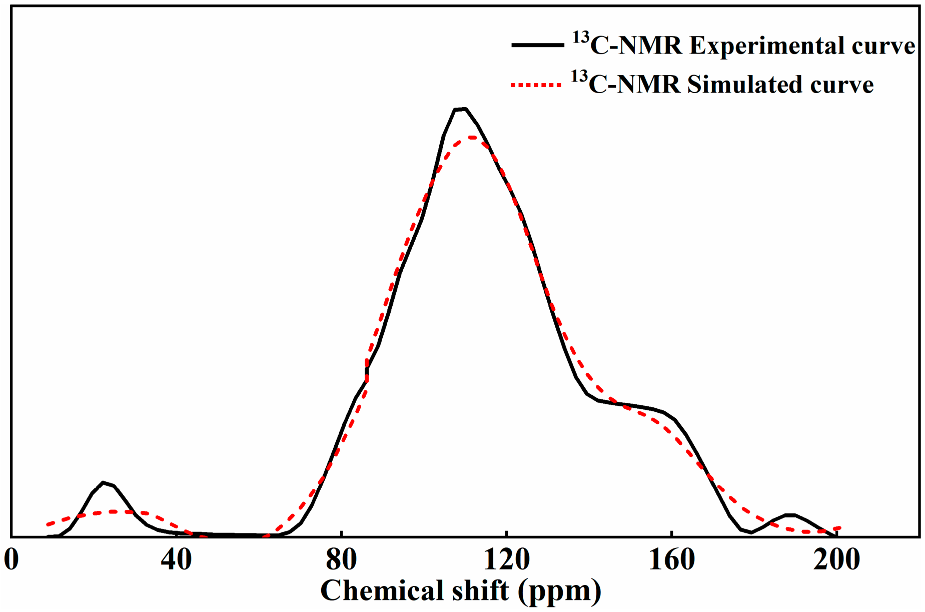

Figure S7. Comparison of ^{13}C-NMR predicted calculated spectra with experimental spectra.

S5. Pore structure

Table S4. Pore structure parameters of samples

Sample	BET Specific surface area (m^2/g)	Pore volume (cm^3/g)	Average pore size (nm)	Relative specific pore volume (%)		
				Microporous	Mesoporous	Macroporous
Cu-BTC@MWS	1265.74	0.223	6.25	37.1	62.8	0.1
R-Cu-BTC@MWS	134.26	0.078	15.51	10.3	78.5	11.2

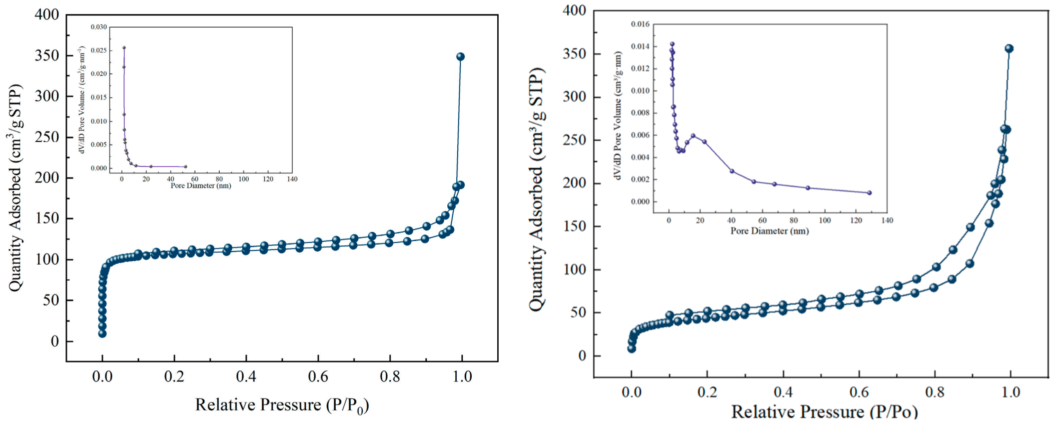

Figure S8. Absorption and desorption curves and pore size distribution of the materials. (a) Cu-BTC@MWS, (b) R-Cu-BTC@MWS.

S6. The results of mercury adsorption energies on Cu-BTC@MWS

Surface electrostatic potential analysis of the Cu-BTC@MWS molecular structure model is presented in Figure S9. The study reveals that sites exhibiting elevated electrostatic potentials on the Cu-BTC@MWS molecular surface are uniformly distributed around the Cu^{2+} within the Cu-BTC molecule. Consequently, these sites are predicted to serve as adsorption sites for Hg^0. Given that Cu-BTC and MWS are primarily encapsulated via ionic bonds, six representative adsorption sites were constructed based on the molecular structures grown around the Cu atom, involving organic ligands such as multimetallic clusters, carbon frameworks, nitrogen atoms, and oxygen-containing functional groups, in conjunction with the electrostatic potential map of Cu-BTC@MWS. Adsorption sites A and B both belong to the reaction sites containing oxygen-containing functional groups, specifically the reaction sites where the carboxyl and aldehyde groups contained in MWS connect with the Cu ion. Adsorption sites C and D represent reaction sites for the doped metals Cu and Fe, respectively. Adsorption sites E and F constitute reaction sites formed by the mutual bonding of C atoms in MWS with O ions in the Cu-BTC organic ligand and metallic Cu ions. The six constructed reaction sites encompass all adsorption reaction types involved in the Hg^0 removal process through cooperative coupling in Cu-BTC@MWS.

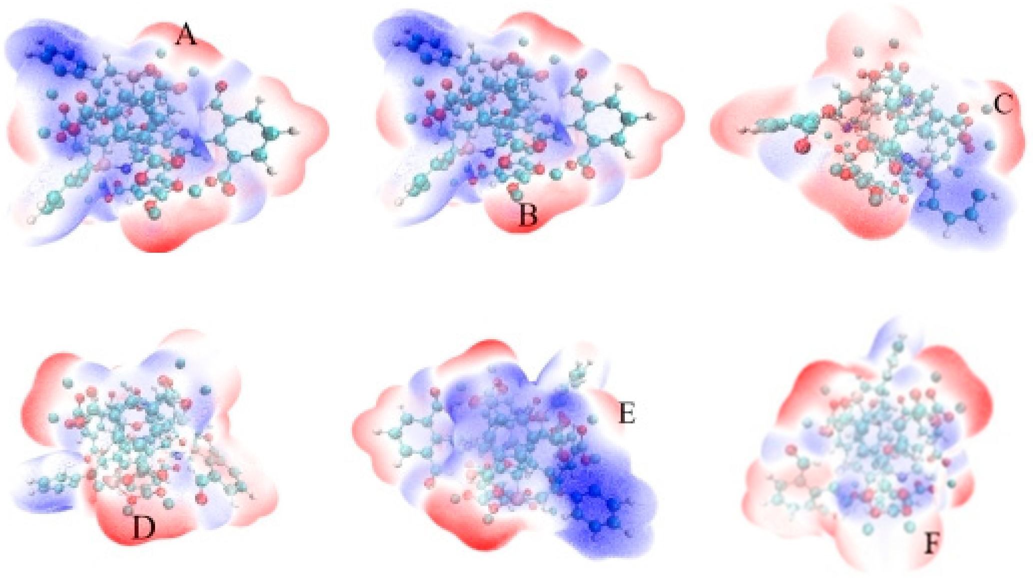

Figure S9. Molecular surface electrostatic potential of Cu-BTC@MWS

The adsorption process of elemental mercury at six constructed adsorption sites was investigated using density functional theory. In all cases, the Hg atom was positioned at the apex of the adsorption site, i.e., the top site. The pre- and post-adsorption configurations are illustrated in Figure 10. Corresponding reaction adsorption energies, adsorption heights, and bond orders were obtained through calculation, as summarized in Table S5.

The relevant atoms within the composite adsorbent are designated as follows: the two Cu ions adjacent to and bonded with the carboxyl group are labeled Cu^{2+}(1) and Cu^{2+} (2) (Figure S10(a)); the Cu ion connected to the oxygen of the aldehyde group is labeled Cu^{2+} (3); around the Fe^{3+} introduced by MWS, the Cu ion in the Cu-BTC molecule connected to the Fe-O bond is labeled as Cu^{2+} (4) (Figure S10 (b)). Similarly, for the Cu^{2+} doped into MWS, the two adjacent Cu ions belonging to the Cu-BTC molecule are labeled Cu^{2+} (5) and Cu^{2+} (6), with the oxygen atom connected to Cu^{2+} (5) labeled as O(1) (Figure S10(c)). Cu ions coupled with both the biochar C(2) atom and the carboxyl oxygen in the Cu-BTC material are labeled Cu^{2+} (7) and Cu^{2+} (8) (Figure S10 (e)). The two Cu^{2+} ions bound to the biochar C(3) atom are labeled Cu^{2+} (9) and Cu^{2+} (10) (Figure S10 (f)). Additionally, the Cu atoms originally doped in MWS are labeled Cu(0), with the C atoms connected to Cu(0) labeled C(1) and C(4), respectively.

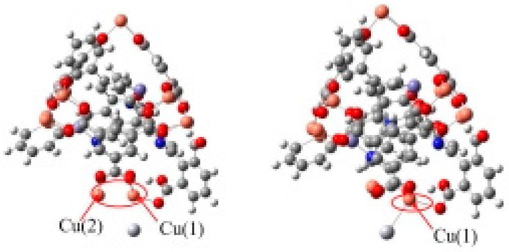

Before After

(a) Adsorption configuration A

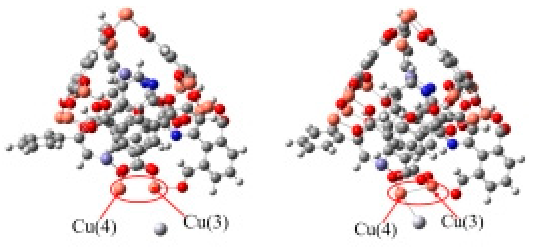

Before After

(b) Adsorption configuration B

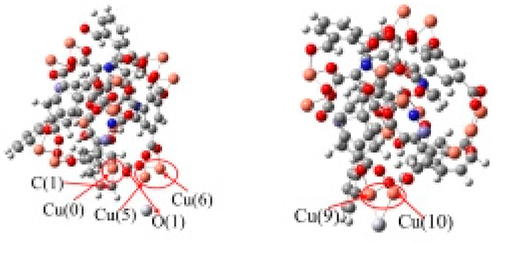

Before After

(c) Adsorption configuration C

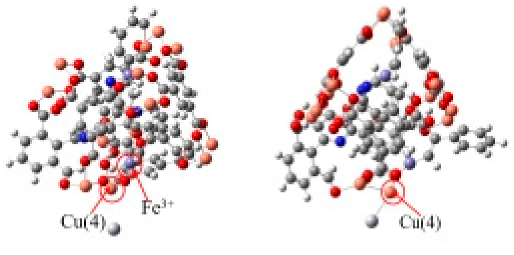

Before After

(d) Adsorption configuration D

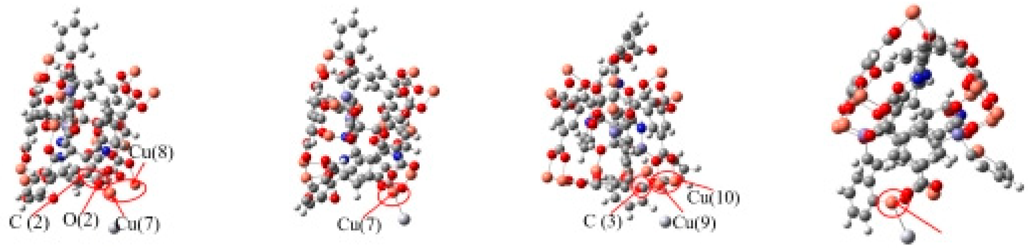

Before After Before After

(e) Adsorption configuration E (f) Adsorption configuration F

Figure S10. Adsorption configurations for the adsorption of Hg^0 at different active sites on the surface of Cu-BTC@MWS

Research shows that all adsorption sites within the composite adsorbent have Hg^0 adsorption energies exceeding 42 kJ/mol, indicating chemical adsorption. This is a significant improvement over pure Cu-BTC material (159.394 kJ/mol) and MWS (98 kJ/mol). It also enables stable retention of adsorbed products on the reaction surface. This suggests that MWS significantly enhances the efficiency of the adsorption system for Hg^0, with adsorption site A (linked to carboxyl groups) exhibiting stronger adsorption than site B (aldehyde groups). For sites created by metal doping, the Cu atom at site C exhibited the highest absolute adsorption energy for Hg^0 at 247 kJ/mol, far surpassing site D (Fe^{3+}) and other sites. Its bond order nearly reached 1, with a bond length of just 2.36 Å, indicating optimal adsorption performance. Additionally, the overall molecular structure of the composite adsorbent undergoes significant changes before and after adsorption. Both adsorption sites E and F have absolute adsorption energies for Hg^0 over 200 kJ/mol, demonstrating relatively good adsorption effects.

Table S5 Adsorption parameters of Hg^0 on the surface of Cu-BTC@MWS under different configurations.

Adsorption configuration	Adsorption energy /(kJ/mol)	Adsorption height /Å		Key-level	
A	-208.192	2.65		0.66	
B	-174.437	2.72		0.59	
D	-172.429	2.82		0.52	
E	-202.942	2.59		0.62	
F	-201.930	2.63		0.61	
C	-247.007	Cu(9)-Hg	Cu(10)-Hg	Cu(9)-Hg	Cu(10)-Hg
		2.36	2.37	0.71	0.68
